# Cell cycle perturbation induced by gemcitabine in human tumor cells in cell culture, xenografts and bladder cancer patients: implications for clinical trial designs combining gemcitabine with a Chk1 inhibitor

**DOI:** 10.18632/oncotarget.18834

**Published:** 2017-06-28

**Authors:** Ryan Montano, Nadeem Khan, Huagang Hou, John Seigne, Marc S. Ernstoff, Lionel D. Lewis, Alan Eastman

**Affiliations:** ^1^ Department of Molecular and Systems Biology, Geisel School of Medicine at Dartmouth, Lebanon, NH, USA; ^2^ Department of Radiology, Geisel School of Medicine at Dartmouth, Lebanon, NH, USA; ^3^ Department of Medicine, Geisel School of Medicine at Dartmouth, Lebanon, NH, USA; ^4^ Norris Cotton Cancer Center, Geisel School of Medicine at Dartmouth, Lebanon, NH, USA; ^5^ Department of Medicine, Roswell Park Cancer Institute, Buffalo, NY, USA

**Keywords:** gemcitabine, S phase arrest, cell cycle checkpoint, Chk1, bladder cancer

## Abstract

Gemcitabine irreversibly inhibits ribonucleotide reductase and induces S phase arrest but whether this occurs in tumors in mice or patients has not been established. Tumor cells in culture were incubated with gemcitabine for 6 h to approximate the administration schedule in a patient. Concentrations that induced persistent S phase arrest thereafter correlated with cell killing. Administration of gemcitabine to mice also demonstrated a persistent S phase arrest in their tumor. The minimum dose that induced almost complete S phase arrest after 24 h (40 mg/kg) was well below the maximum tolerated dose in mice. S phase arrest was also observed in tumors of bladder cancer patients receiving gemcitabine. The Chk1 inhibitor MK-8776 sensitized cells to gemcitabine with the greatest cell killing when added 18 h after gemcitabine. In mice, the administration of MK-8776 18 h after gemcitabine elicited positivity for the DNA damage marker γH2AX; this also occurred at relatively low dose (40 mg/kg) gemcitabine. Hence, in both cell culture and xenografts, MK-8776 can markedly enhance cell killing of cells reversibly arrested in S phase by gemcitabine. Some cell lines are hypersensitive to MK-8776 as monotherapy, but this was not observed in xenograft models. Effective monotherapy requires a higher dose of Chk1 inhibitor, and target inhibition over a longer time period as compared to its use in combination. These results have important implications for combining Chk1 inhibitors with gemcitabine and suggest that Chk1 inhibitors with increased bioavailability may have improved efficacy both in combination and as monotherapy.

## INTRODUCTION

Gemcitabine is used as monotherapy or in combination for treatment of patients with bladder, pancreas, ovary, breast and non-small cell lung cancer. The most common administration schedule is gemcitabine at 1000 mg/m^2^ infused intravenously over about 30 min on days 1 and 8 of a 21-day cycle, although many variations exist. Lower doses and more frequent administration schedules have been suggested but have not gained general acceptance. While the mechanisms of action of gemcitabine have been extensively studied in cell culture, few if any pharmacodynamic studies have been performed in patients to determine whether the *in vitro* defined mechanisms have relevance to the *in vivo* drug action.

DNA damaging drugs such as gemcitabine induce cell cycle arrest in S or G_2_ phase in a manner regulated by Chk1 [1]. The arrest permits time for DNA repair before the cell progresses through the cell cycle. Chk1 inhibitors (Chk1i) can abrogate arrest permitting cells to progress through the cell cycle before they are able to repair the initial damage to DNA. Additionally, Chk1 stabilizes stalled replication forks such that Chk1i cause replication fork collapse. In both cases, Chk1i enhances DNA double-strand breaks and increases tumor cell killing. At least four Chk1i have entered clinical trials, particularly in combination with gemcitabine, but the therapeutic response to date has not been impressive [2–5]. Here, we provide a detailed pharmacology study of gemcitabine in cell culture, mice and man, and assess the impact of combining gemcitabine with the Chk1i MK-8776. In addition, we have previously noted that some cancer cell lines are hypersensitive to MK-8776 as a single agent [6]. Our observations provide a foundation to further develop Chk1i as both monotherapy and in combination with gemcitabine.

Gemcitabine (difluorodeoxyctidine; dFdC) has a relatively short terminal plasma half-life (42-94 min), but following transport across a cell membrane it undergoes anabolic phosphorylation initially by deoxycytidine kinase and then to dideoxynucleotides (dFdCDP) and trideoxynucleotides (dFdCTP) whose intracellular half-lives can be as long as 20 h (gemcitabine package insert). dFdCTP is incorporated into DNA while dFdCDP irreversibly inhibits ribonucleotide reductase thereby starving cells for deoxyribonucleotides. The relative importance of each of these pathways remains to be resolved. Both pathways cause replicative stress that activates Chk1 to stabilize the replication fork and prevent further replication on damaged DNA. If gemcitabine worked primarily through incorporation into DNA, then incubation with a Chk1 inhibitor (Chk1i) would abrogate S phase arrest, allowing cells to proceed through S into M and into premature mitosis, as seen with many other DNA damaging agents [7, 8]. Alternately, if the primary target is ribonucleotide reductase, then addition of Chk1i would fail to induce S phase progression because of the absence of dNTPs. Our prior results and those presented here clearly demonstrate that Chk1i induces replication fork collapse and DNA double-strand breaks in S phase cells without S phase progression, consistent with the inhibition of ribonucleotide reductase being the primary mechanism. However, this observation does not rule out the possibility that incorporation into DNA is occurring concurrently. There is an important caveat if both pathways occur: the concurrent increase in dFdCTP and decrease in dCTP has been proposed to increase dFdCTP incorporation into DNA, an action known as self-potentiation [9]. However, the incorporation of dFdCTP into DNA requires ongoing DNA replication and the presence of normal deoxyribonucleotides, which would be limited when ribonucleotide reductase is inhibited. Hence, the extent of incorporation of dFdCTP into DNA would also be self-limiting because of the lack of other dNTPs.

Considering that gemcitabine is generally administered to patients as a short intravenous infusion (30 min), and has a short half-life, continuous exposure of cells to gemcitabine *in vitro*, as commonly studied, has little relevance to the clinical administration. However, as ribonucleotide reductase is irreversibly inhibited, the impact of gemcitabine persists long after the drug has been removed. For all the cell culture experiments presented here, we have used a nominal 6 h incubation with gemcitabine, followed by its removal, and have studied cell cycle arrest and replication fork collapse thereafter. This current study was designed to better understand the cell cycle perturbation induced by gemcitabine in cell culture, animal models and patients with cancer, and to provide rationale suggestions to facilitate improved schedules for combining gemcitabine with a Chk1i.

## RESULTS

### Cytotoxicity of gemcitabine

**Figure 1:**
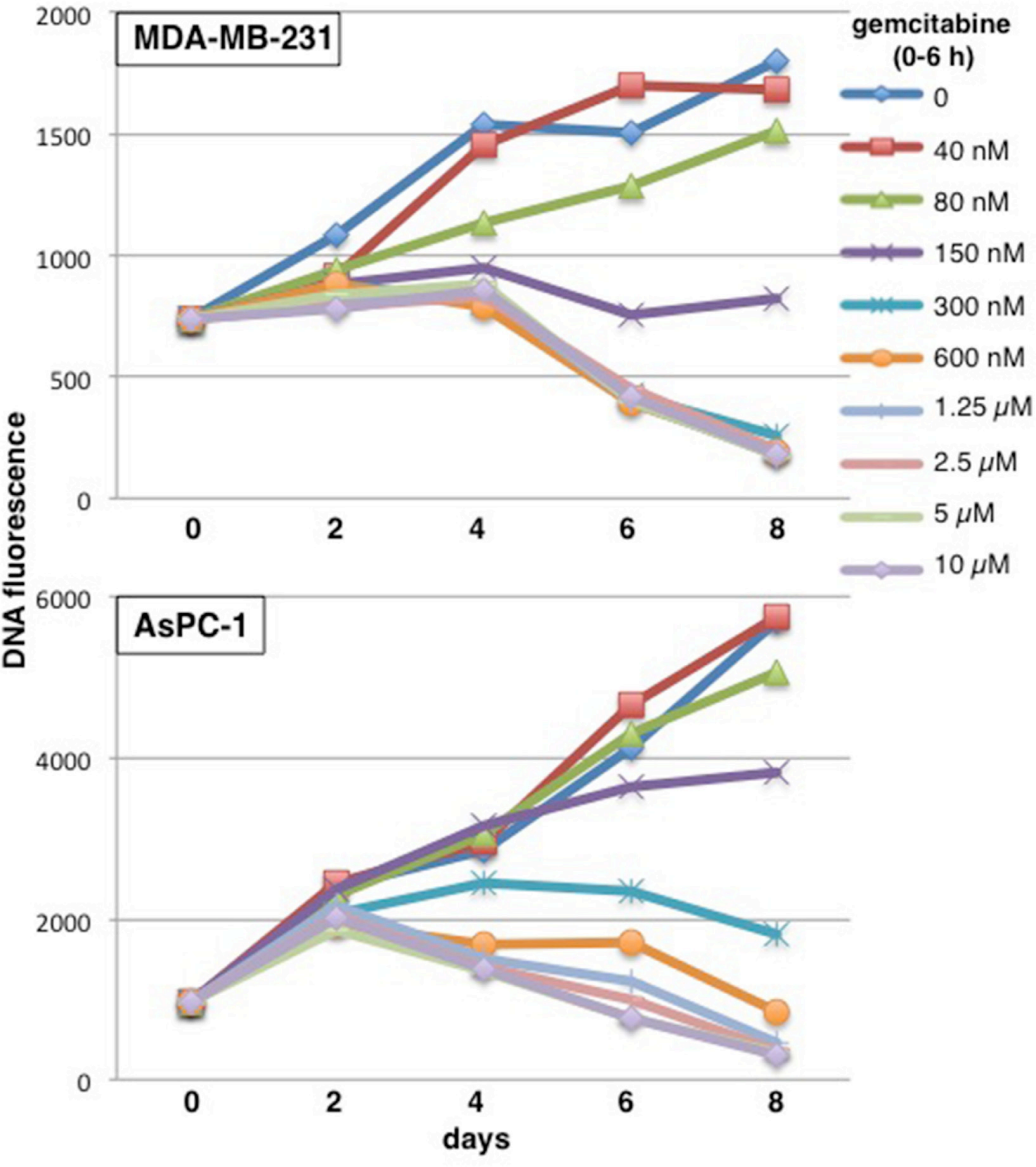
Concentration and time dependent cytotoxicity induced by gemcitabine in MDA-MB-231 and AsPC-1 cells Cells were plated at 10,000 cells/well in a 96 well plate. The following day, 8 wells were incubated with each of the indicated concentrations of gemcitabine for 6 h. Drug was removed and cells incubated in drug-free medium for up to 8 days. Plates were harvested at time zero (before drug) and every 2 days. DNA content was measured as an indication of the number of cells present and the relative fluorescence is reported. Representative growth curves are shown and replicate experiments are shown in Figure 5.

Most cytotoxicity experiments use growth inhibition as an endpoint [often erroneously called a “viability assay” [10]. Our prior experiments also focused on growth inhibition and reported 50% growth inhibition at 60 nM gemcitabine in MDA-MB-231 cells and 115 nM gemcitabine in AsPC-1 cells (assessed 6 days after a 6-h incubation with gemcitabine) [11]. However, the goal in treating patients is not just to inhibit cell growth but to kill cells and thus reduce the size of the tumor. Here, we have performed more extensive cytotoxicity assays over a longer time frame with a modified assay to assess potential cell killing. This assay requires that a higher number of cells are plated in a 96 well plate format, incubated with gemcitabine for 6 h, then harvested every 2 days. Relative cell number was assessed by quantifying DNA. One plate was harvested at time zero to provide an assessment of the starting inoculum, and thereby permit an assessment of whether cell number decreased over time. Because of the high starting inoculum, untreated cells slow rapidly as the well becomes saturated, and may eventually detach such that there is an apparent decrease in the DNA content. Cells whose growth has been slightly inhibited quickly attain the same value as control cells. Hence, this modification is relatively uninformative at low concentrations of drug. However, clear differences are observed at higher drug concentrations. MDA-MB-231 cells incubated with 150 nM gemcitabine from 0 – 6 h showed a slight increase in the fluorescent signal over the first few days probably as a consequence of cells accumulating in S and G_2_ (because the assay is recording DNA content), but no further increase in cells over 8 days (Figure 1). Higher concentrations showed a marked decrease in cells, but it is important to note that this cell death was delayed and not evident until day 6. Furthermore, very high concentrations did not kill cells any quicker. Similar results were observed in AsPC-1 cells with stable cell number over the time course observed following incubation with 300 nM gemcitabine (Figure 1).

### Cell cycle arrest and recovery following gemcitabine treatment in cell culture

**Figure 2:**
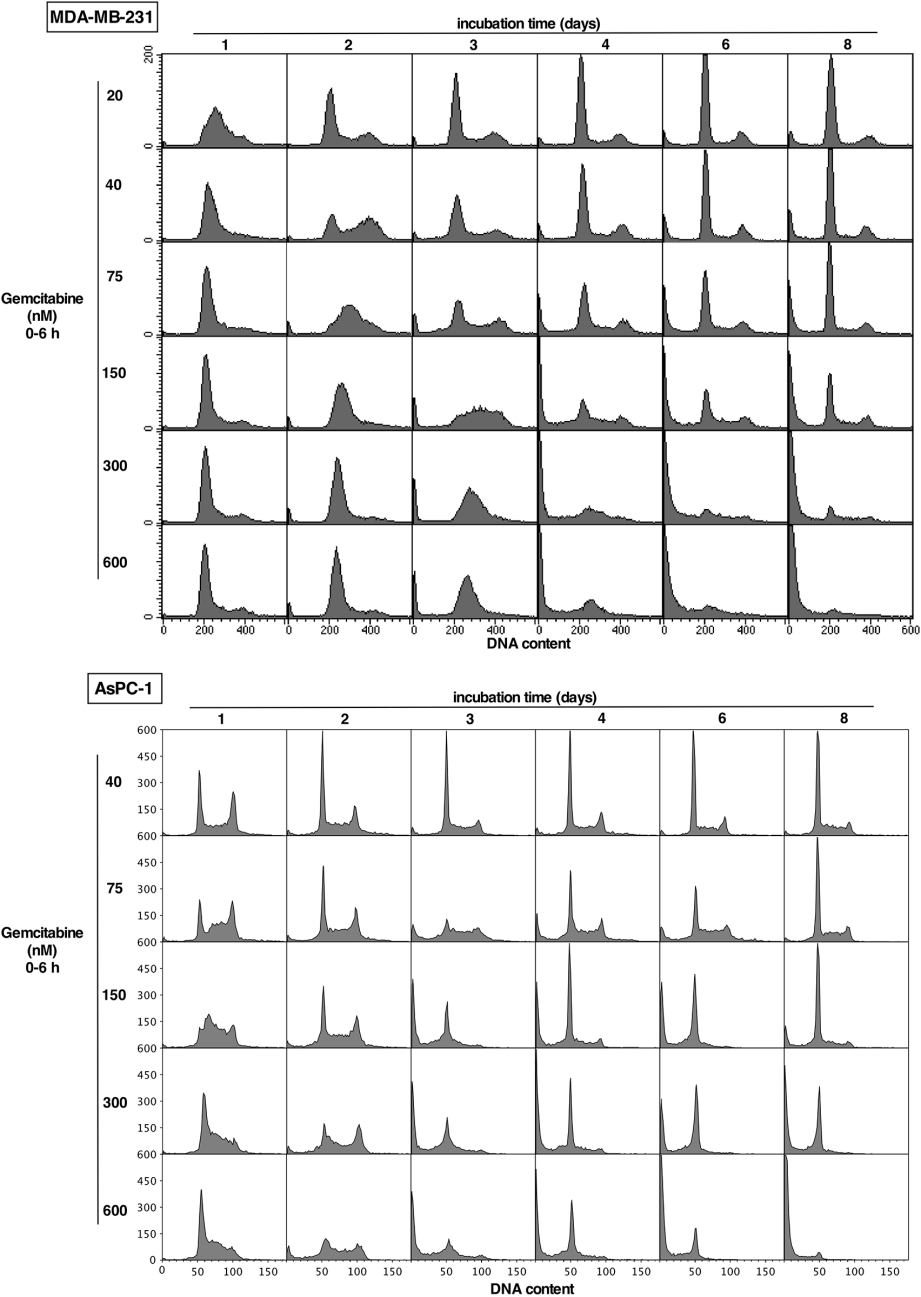
Impact of gemcitabine on cell cycle perturbation in MDA-MB-231 and AsPC-1 cells Each cell line was incubated with the indicated concentration of gemcitabine for 6 h, then the drug was removed and cells incubated in drug-free medium for up to 8 days. Attached and detached cells were harvested and pooled at the indicated times, and analyzed for DNA content by flow cytometry. Replicate experiments for the lower concentrations of gemcitabine in MDA-MB-231 cells for up to 72 h have previously been published [11].

Cell cycle perturbation was analyzed over the concentration and time range of the cytotoxicity assays in Figure 1. In MDA-MB-231 cells, a low concentration of gemcitabine (20 nM) induced arrest in mid-S phase at 24 h, and the cells appeared to have fully recovered by 48 h (Figure 2). Higher concentrations induced arrest in early S phase (very early S at high concentrations that appears similar to G_1_). The rate of recovery depended on concentration. Following 150 nM gemcitabine, the majority of cells persisted in S phase for 3 days, some subsequently died (sub-G_1_) while others recovered, consistent with the overall lack of growth over 8 days. At higher concentrations the cells still progressed into early S phase but did not recover, with the majority having died by 6 days. These results suggest that cells can tolerate arrest in S phase for several days, but prolonged arrest is lethal.

Similar experiments in AsPC-1 cells showed a slightly different pattern of recovery and death (Figure 2). Again at the lower concentrations, cells recovered from the S phase arrest that was apparent after 1 day. However, the cells did not demonstrate prolonged S phase arrest at higher concentrations; they possibly died more rapidly (sub-G_1_ cells), while survivors appeared to persist in G_1_ (apparently post-mitotic), but even these cells died eventually at the highest concentration.

### The effect of gemcitabine in xenograft tumor models

We extended the *in vitro* analysis to an *in vivo* situation to assess the dose and time at which cell cycle arrest occurs in tumors following administration of gemcitabine to mice. Geminin is a marker of S and G_2_ cells as it is proteolytically degraded in M and G_1_. As regions of proliferation can vary across the tumor based on nutrient and oxygen availability, we concurrently scored for Ki67 which is expressed at all phases of the cell cycle except G_0_. Hence the ratio of geminin/Ki67 represents the proportion of proliferating cells that are in S phase. MDA-MB-231 cells only provide a very narrow margin of proliferation at the periphery of the tumor that was not very amenable to analysis. However, AsPC-1 tumors provide an excellent distribution of proliferating cells throughout much of the tumor, and we have already reported tumor growth delay in this model. Specifically, 150 mg/kg gemcitabine i.p. induced close to tumor stasis when administered to mice once per week over three weeks, whereas addition of MK-8776 18 h after each gemcitabine treatment induced about 25% tumor regression [11].

**Figure 3:**
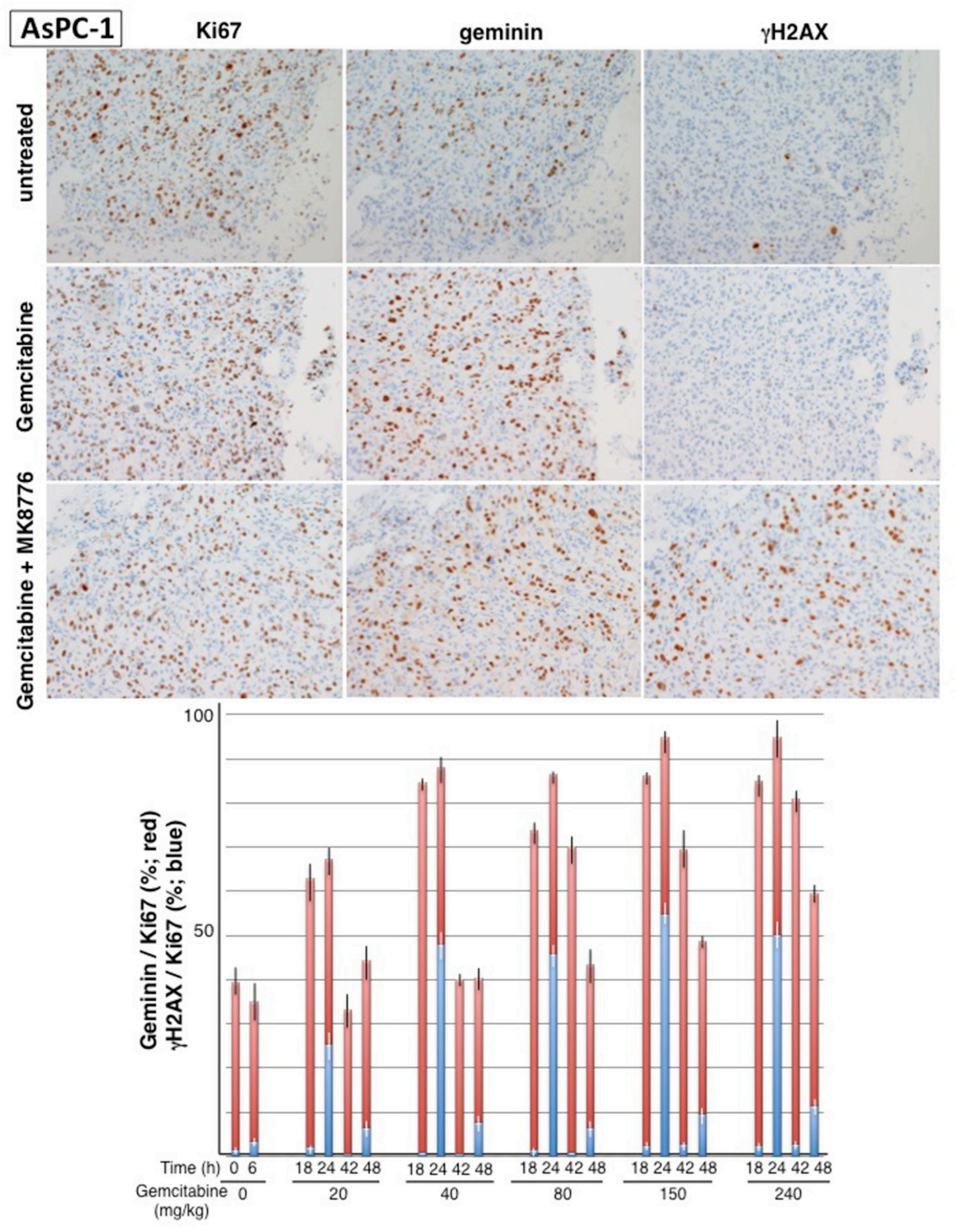
Impact of gemcitabine and MK-8776 on cell cycle perturbation and γH2AX in AsPC-1 pancreas tumor xenografts Tumor bearing-mice were administered 20 – 240 mg/kg gemcitabine and tumors harvested at 18 and 42 h. In addition, mice were administered 50 mg/kg MK-8776 at either 18 or 42 h and then tumors harvested at 24 or 48 h, respectively. Serial sections from the tumors were stained for Ki67, geminin and γH2AX; representative tumor sections are shown. Results reflect the percent of Ki67-positive cells that are positive for geminin (red) or γH2AX (blue). Results represent the mean and SEM for at least 2 sections from 2-4 mice at each condition.

Mice were injected with 20 – 240 mg/kg gemcitabine and tumors resected at 18 and 42 h for analysis of geminin, Ki67 and γH2AX, a common marker for DNA damage (Figure 3). Additional mice were treated with MK-8776 at either 18 or 42 h, and then tumors resected 6-h later; these results are discussed in a following section. In untreated tumors about 40% of the Ki67-positive cells were also positive for geminin. By 18 h after administration of gemcitabine, this value increased to >80% at all doses except the lowest (20 mg/kg). The notable difference between the mice treated with different doses was the rate at which the tumors recovered from the arrest with full recovery by 42 h after 40 - 80 mg/kg but more persistent arrest at the higher concentrations. Consequently, treatment with gemcitabine at 40 mg/kg mimics 40-75 nM *in vitro*, both of which cause transient cell cycle arrest. Treatment of mice with 150 mg/kg gemcitabine is comparable to the 150-300 nM treatment *in vitro*, and is also consistent with the dose that caused tumor stasis in our prior experiments [11]. Very few γH2AX -positive cells were observed in any cohort following treatment with gemcitabine alone.

### The effect of gemcitabine in human tumors

**Figure 4:**
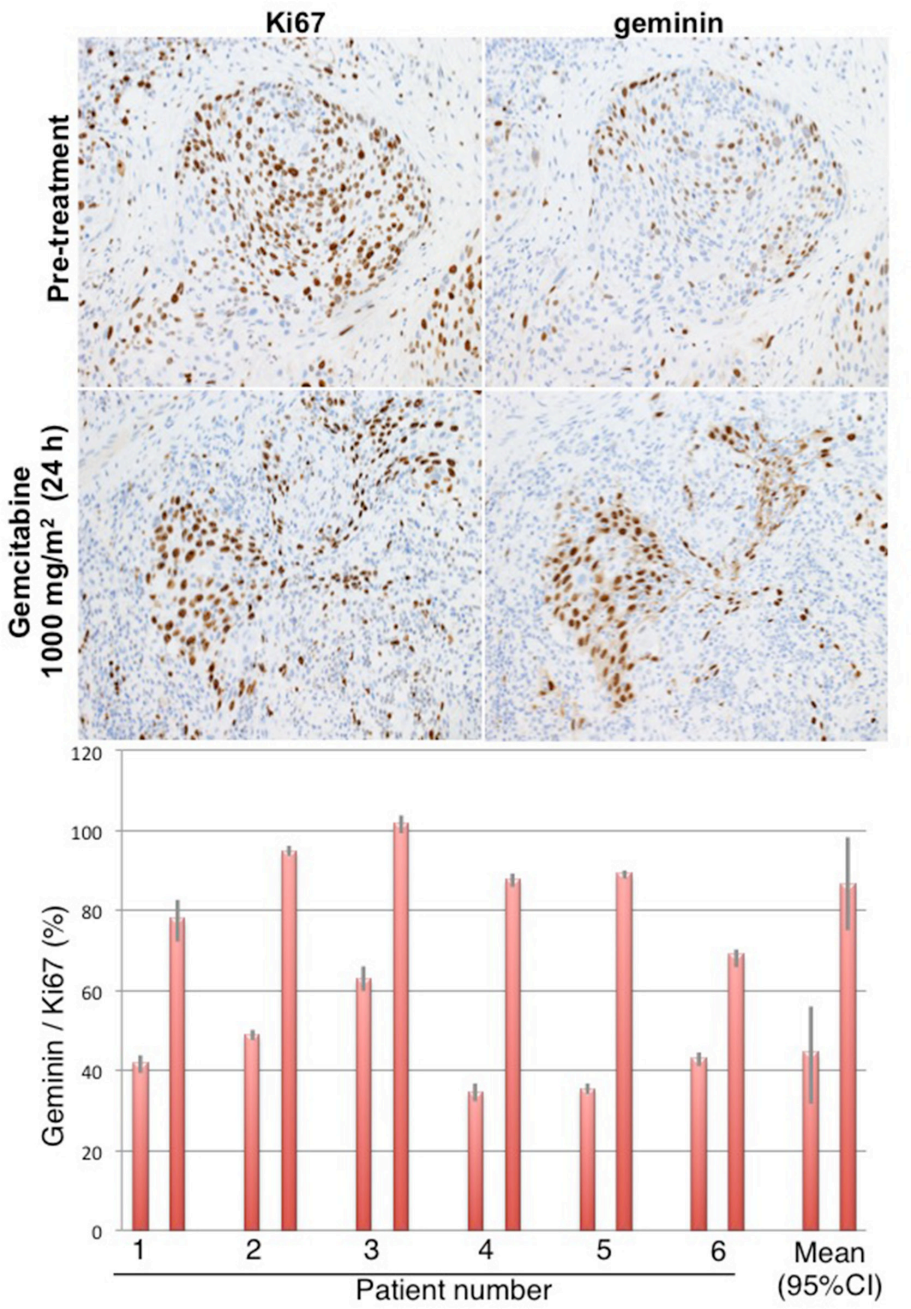
Cell cycle perturbation induced in bladder tumors of patients administered gemcitabine Patients were administered 1000 mg/m^2^ gemcitabine, and their tumor was resected approximately 24 h later. Serial sections were stained for Ki67 and geminin, and results expressed as the percent of Ki67-positive cells that were positive for geminin. Results from 6 individual patients are shown with SEM for at least 4 fields from each biopsy. The mean of all patient data is summarized plus the 95% confidence interval.

To determine whether the observed cell cycle perturbations in xenograft tumors also occur in tumors in patients, we performed a human clinical trial in which bladder cancers were biopsied before gemcitabine (a diagnostic biopsy), and then 24 h after intravenous administration of 1000 mg/m^2^ gemcitabine, the patients underwent a transurethral resection of the bladder tumor as standard-of-care. The pre- and post-therapy tumors were fully evaluable from 6 patients (Figure 4). Prior to therapy, approximately 40% of the Ki67-positive tumor cells stained for geminin. Following therapy, this proportion dramatically increased to ∼80%, and is therefore similar to what was observed in mice. These results demonstrate that administration of gemcitabine to humans causes a similar accumulation of cells in S phase as observed in mice.

### MK-8776 sensitizes cells in culture to cytotoxic effects of gemcitabine

Our interest in the cell cycle perturbation following administration of gemcitabine derives from our studies with the Chk1i MK-8776. We previously demonstrated that the greatest inhibition of cell growth occurred when a pulse of MK-8776 was added 18-24 h after gemcitabine (which was added from 0-6 h) [11]. The 6-h pulse of MK-8776 was based on its known pharmacokinetics in patients and reflected the time over which MK-8776 exceeded a concentration of 1 μM in plasma [12]. The primary reason for the sensitivity of this schedule is that 18-24 h is the time at which the majority of cells have arrested in S phase following gemcitabine, and so are susceptible to Chk1i. The improved efficacy of this delayed administration of Chk1i following gemcitabine was also confirmed in human xenograft tumor models [11]. However, only partial tumor regression was observed. In this study we have further investigated the MK-8776-mediated sensitization to gemcitabine.

**Figure 5:**
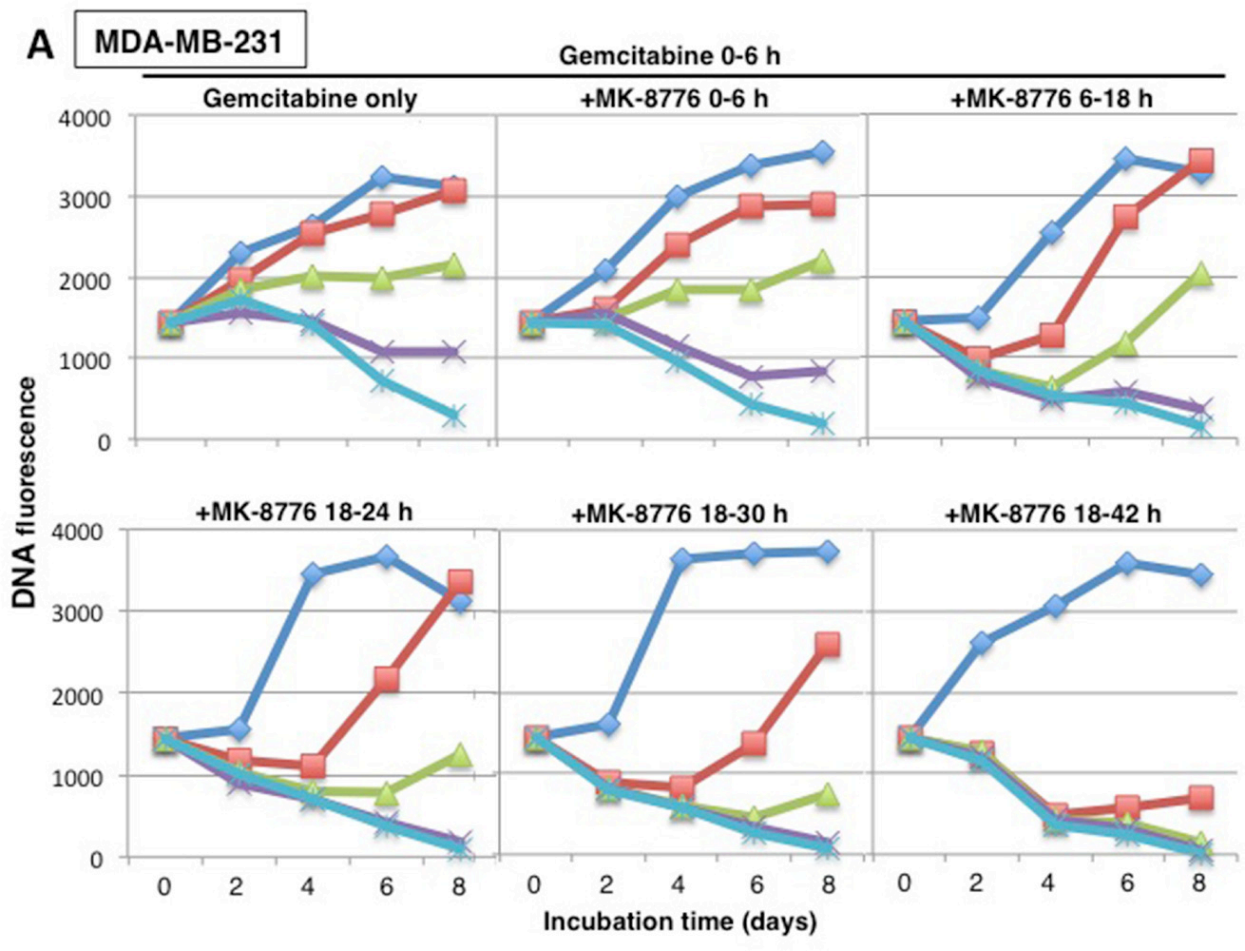

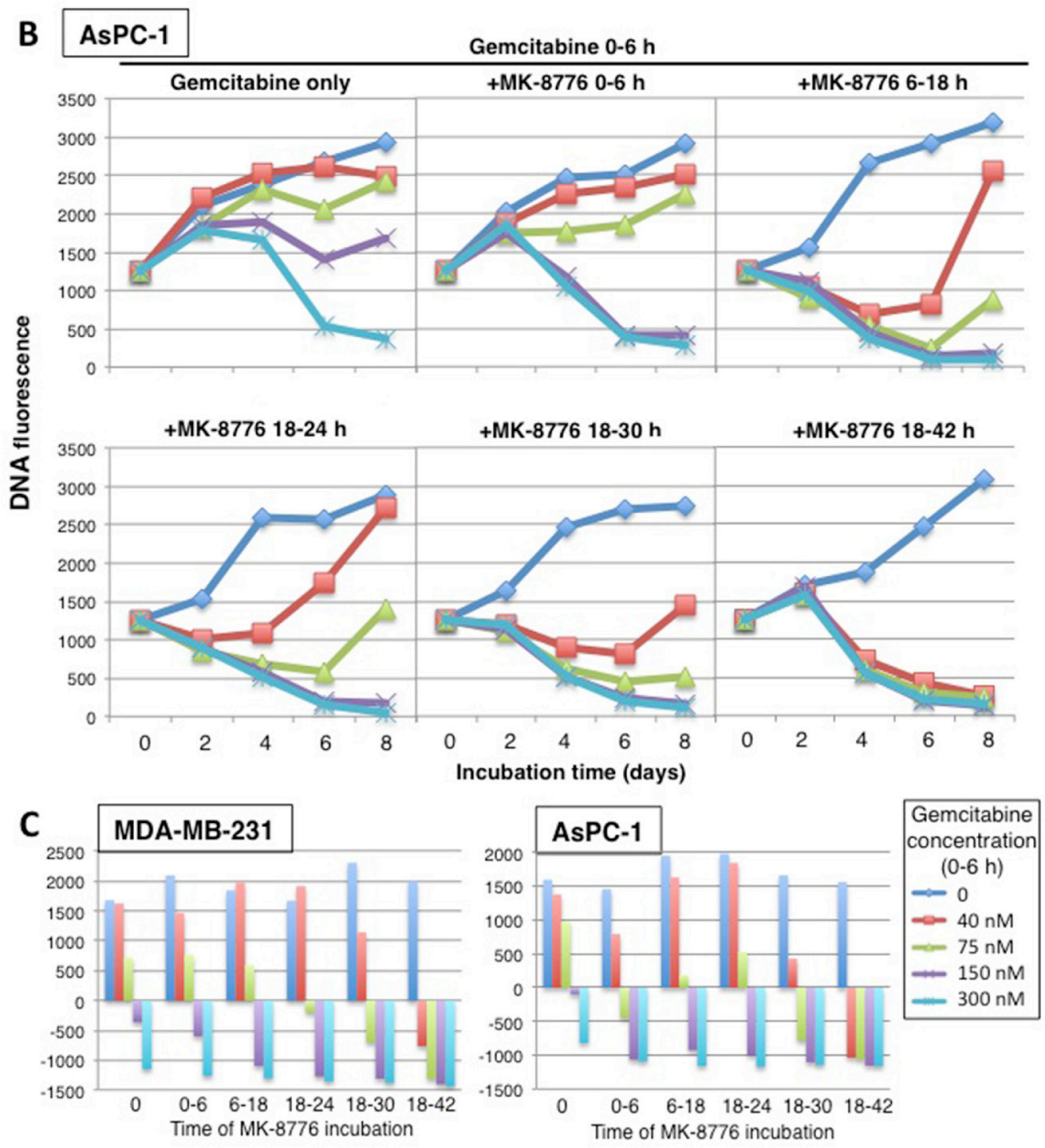
Concentration and time dependent cytotoxicity induced by gemcitabine plus MK-8776 **(A)** MDA-MB-231 and **(B)** AsPC-1 cells were subjected to cytotoxicity assays as performed in Figure 1 with 0 – 300 nM gemcitabine added from 0-6 h, but with 1 μM MK-8776 added at various different times and for different periods. Cells were harvested for up to 8 days and DNA fluorescence assayed as an indication of the number of cells present. The experiment was repeated two times; representative growth curves are shown and a replicate experiment for the first panel is shown in Figure 1. **(C)** The data at day 8 are also plotted as a waterfall plot with the zero value representing the fluorescence at day 0. These plots represent the average values of two independent experiments.

Building on the modified cytotoxicity approach presented in Figure 1, we incubated MDA-MB-231 cells with various concentrations of gemcitabine for 6 h, then added MK-8776 at various times thereafter and for various time periods, harvesting cells every 2 days up to day 8 (Figure 5A). As expected, concurrent incubation with MK-8776 had little impact on cytotoxicity compared to gemcitabine alone with 150 nM gemcitabine exhibiting no growth; this can be considered equivalent to “stable disease.” In contrast, MK-8776 added from 18-24 h markedly increased cytotoxicity, with transient “regression” observed even at 40 nM gemcitabine. We questioned whether longer incubations with MK-8776 would further enhance cytotoxicity. Addition of MK-8776 from 18-30 h further enhanced cytotoxicity while incubation from 18-42 h caused almost complete “regression” even in cells incubated with 40 nM gemcitabine. This experimental approach was repeated in AsPC-1 cells with very similar results (Figure 5B). The results are also summarized as a waterfall plot reflecting the growth or regression observed after 8 days (Figure 5C). These results confirm the value of delaying the addition of MK-8776, but also show that a longer incubation time provides even greater cytotoxicity.

**Figure 6:**
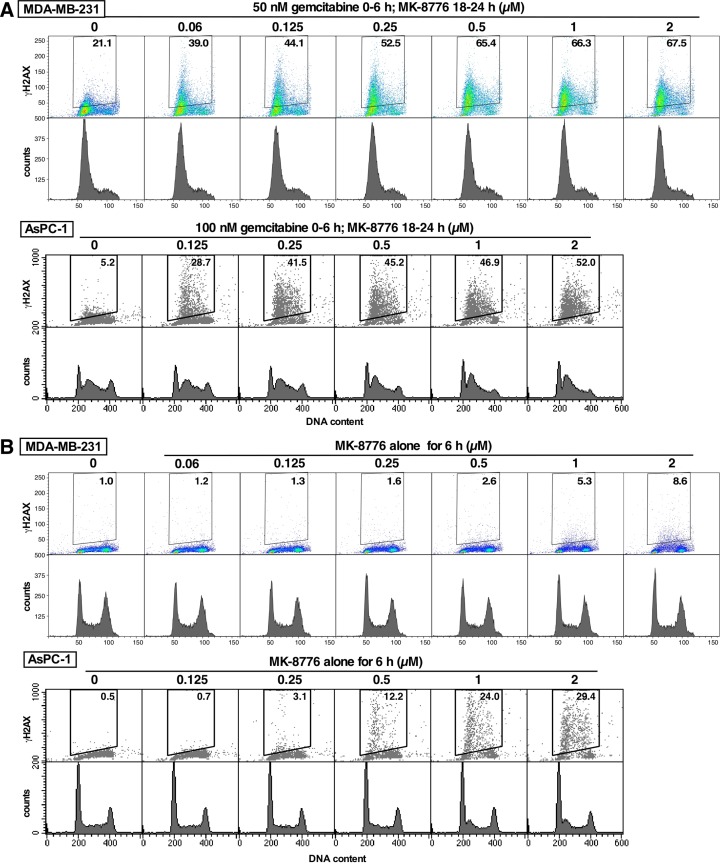
Comparison of the MK-8776 concentrations required to induce γH2AX either as a single agent or following S phase arrest induced by gemcitabine **(A)** MDA-MB-231 (top) and AsPC-1 cells (bottom) were incubated with 50 or 10 nM gemcitabine, respectively, for 6 h. Fresh media was added and 0-2 μM MK-8776 was added from 18-24 h. Cells were then harvested and analyzed by 2-dimensional flow cytometry for γH2AX and DNA content. **(B)** The cell lines were incubated with 0-2 μM MK-8776 for 6 h then harvested and analyzed by flow cytometry for γH2AX and DNA content. The experiments have been repeated with similar results; a few conditions are replicated in Figure 7.

Incubation of both MDA-MB-231 and AsPC-1 cells in culture with gemcitabine caused only a slight increase in γH2AX but this was markedly increased upon addition of MK-8776 (Figure 6A). This experiment was performed at a concentration of gemcitabine that caused arrest of the cells in early S phase. The concentration of MK-8776 added from 18-24 h after gemcitabine was varied to assess its efficacy in this model (and to compare to MK-8776 as monotherapy discussed below). MK-8776 at 0.1 μM caused extensive γH2AX, which reached a maximum around 0.5 μM. Importantly, the addition of MK-8776 did not cause progression of the cells through S phase, rather the γH2AX occurred at the same point of the cell cycle at which they were arrested by gemcitabine consisted with collapse of replication forks.

### Combination of gemcitabine and MK-8776 in xenograft tumor models

The induction of γH2AX upon addition of MK-8776 (Figure 6) provided the rationale for investigating the appearance of γH2AX in tumors. Mice bearing AsPC-1 xenografts were injected i.p. with gemcitabine and then with 50 mg/kg MK-8776 after an additional 18 or 42 h; tumors were harvested 6 hours later (Figure 3). This had little impact on the geminin/Ki67 ratio but dramatically increased the percent of cells that were positive for γH2AX; by 24 h, up to 50% of Ki67-positive cells were also positive for γH2AX. Addition of MK-8776 at 42 h had less impact with only about 10% positive for γH2AX, suggesting the cells are already recovering from the gemcitabine-mediated replicative stress at this time.

### MK-8776 as a single agent

Administration of MK-8776 alone to mice bearing AsPC-1 xenografts had little impact on induction of γH2AX in the tumor (Figure 3). Furthermore, MK-8776 alone had no impact on growth of these tumors in mice [11]. This is an important observation because AsPC-1 is one of the cell lines most sensitive to MK-8776 as a single agent [6]. To explain this discrepancy, we first compared the efficacy of MK-8776 to induce γH2AX either in combination with gemcitabine or as a single agent in cell culture. AsPC-1 cells arrested in S phase with gemcitabine (0-6 h, analyzed at 24 h) exhibited a slight increase in γH2AX that was markedly increased by incubation with 0.125 nM MK-8776 (18-24 h) (Figure 6). In contrast, when added as a single agent, a 10-fold higher concentration of MK-8776 was required to induce a comparable level of γH2AX over a 6-h period. It is worth noting that more cells are present in S phase after gemcitabine plus MK-8776 treatment, but the results still show a clear need for higher concentrations of MK-8776 when used as a single agent. Similar results were also obtained in MDA-MB-231 cells, except the amount of γH2AX resulting from incubation with MK-8776 alone was far lower (Figure 6) because these cells are relatively resistant to this drug as a single agent [6].

**Figure 7:**
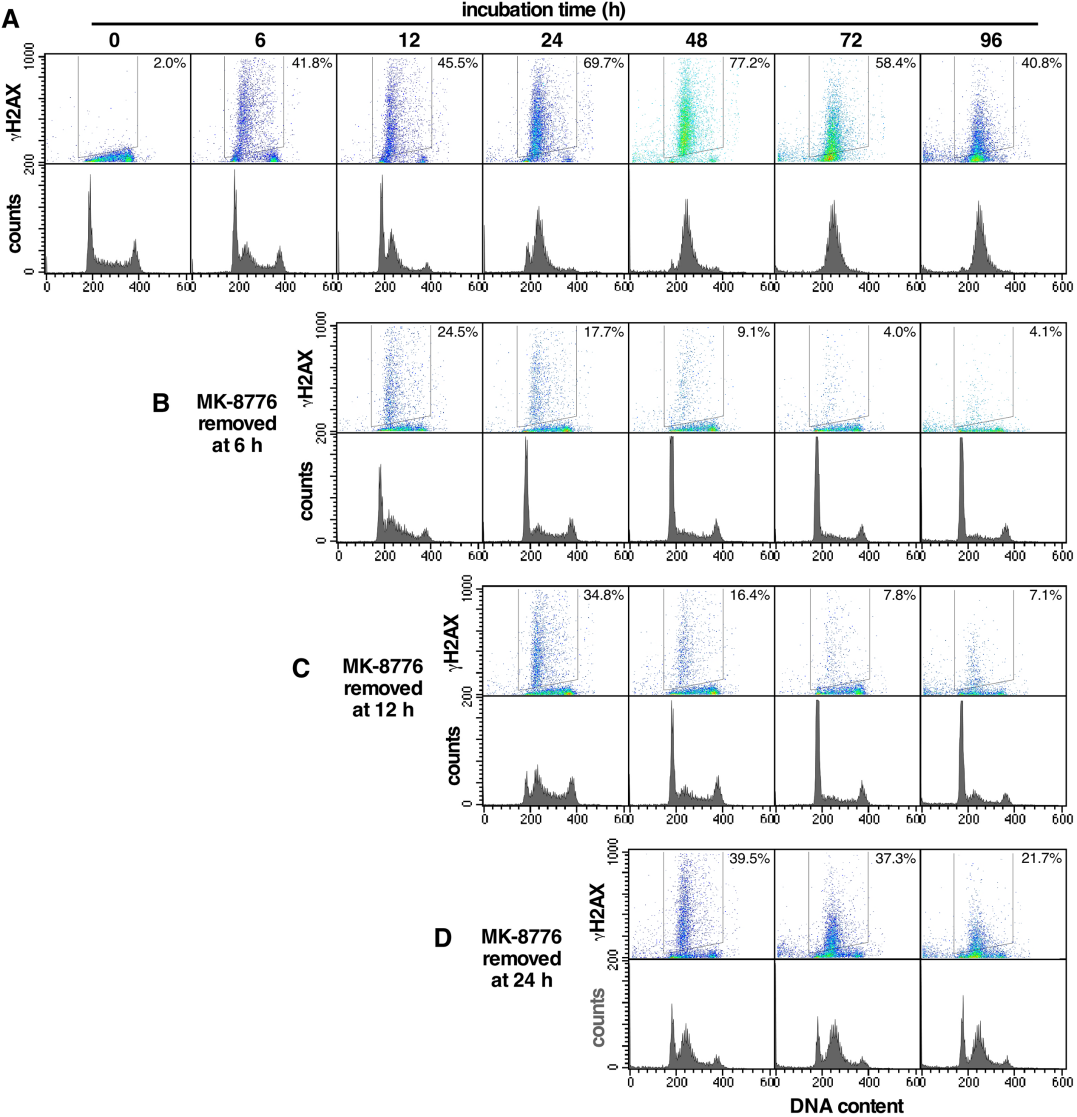
Recovery of AsPC-1 cells from incubation with MK-8776 **(A)** AsPC-1 cells were incubated continuously for up to 96 h with 2 μM MK8776. **(B-D)** MK-8776 was removed after either 6 h **(B)**, 12 h **(C)** or 24 h **(D)** and incubation continued in the absence of drug. Cells were harvested from 0 – 96 h and analyzed by 2-dimensional flow cytometry for γH2AX and DNA content. The percentages represent the γH2AX-positive cells within the indicated gate. The experiments have been repeated with similar results; a few conditions are replicated in Figure 6.

Next, we assessed the ability of AsPC-1 cells to recover from single agent MK-8776 treatment. AsPC-1 cells were incubated continuously with 2 μM MK-8776 for up to 96 h (Figure 7). Most of the cells initially in S phase show rapid accumulation of γH2AX, but as cells continue to enter S, they also accumulate γH2AX. However, when MK-8776 was removed at 6 h, cells in S phase appear to recover and those still in G_1_ did not accumulate γH2AX as they progress into S phase. Recovery of cells after 12 h was also seen, but after 24 h, recovery was much less. These results suggest that an incubation time of at least 24 h is required for MK-8776 to be sufficiently cytotoxic to cells. This likely explains why MK-8776 as a single agent appeared ineffective against xenograft tumors.

**Figure 8:**
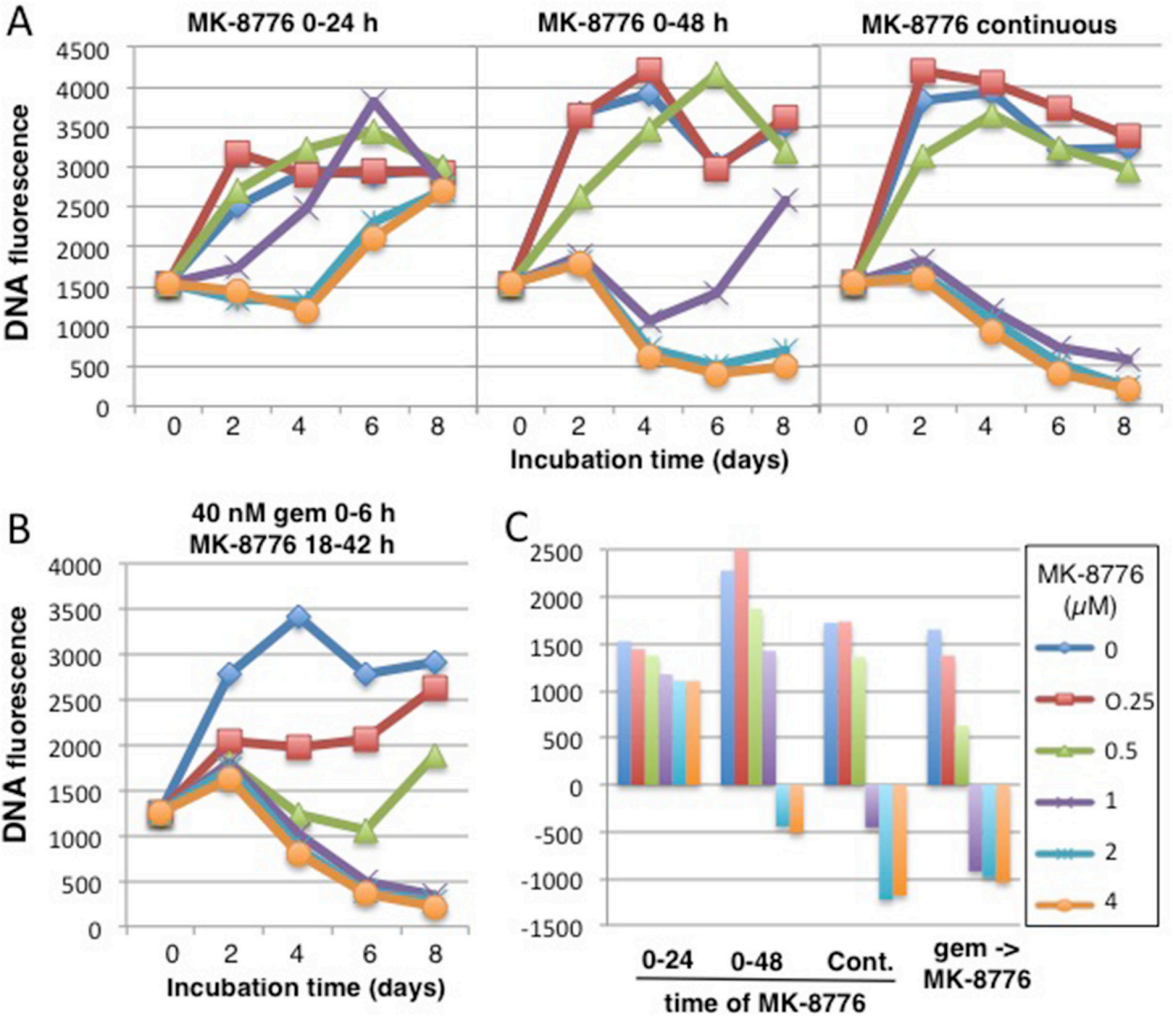
Comparison of the cytotoxicity induced by MK-8776 as a single agent versus its efficacy in combination with gemcitabine **(A)** AsPC-1 cells were incubated with 0.25-4 μM MK-8776 for either 24 h, 48 h or continuously for 8 days, and DNA fluorescence scored every 2 days. **(B)** AsPC-1 cells were incubated with 40 nM gemcitabine from 0-6 h, then with 0.25 – 4 μM MK-8776 from 18-42 h and DNA fluorescence scored every 2 days for 8 days. The experiment was repeated two times with similar results and representative growth curves are shown. **(C)** The data at day 8 are plotted as a waterfall plot with the zero value representing the fluorescence at day 0. These plots represent the average values of two independent experiments.

Finally, we used our modified cytotoxicity assay to compare the potency of MK-8776 either as a single agent or in combination with gemcitabine (Figure 8). A 24-h incubation with 2 μM MK-8776 caused stasis for 4 days, but cells began to recover after this time (likely because some cells had not entered S during this 24-h period; Figure 7). If the incubation time was extended to 48 h, or continuously, there was much greater impact as reflected in the decrease in cell numbers. For comparison, we incubated cells with 40 nM gemcitabine for 6 h, then titrated MK-8776 for a 24-h period starting at 18 h (reiterating conditions in Figure 5). Concentrations of 1 μM MK-8776 or greater caused extensive tumor cell kill over the 8 day time frame. These results reemphasize that lower concentrations of MK-8776 can be effectively combined with gemcitabine than when MK-8776 is used as a single agent. In addition, the incubation with MK-8776 as a single agent needs to be longer than when used in combination.

In a preliminary attempt to determine whether increased MK-8776 exposure would enhance the appearance of γH2AX, we increased both the dose and frequency of administration. MK-8776 at 100 mg/kg administered at 0, 4 and 8 h slightly increased the percent of cells in S/G_2_ phase but only about 25% of the geminin positive cells (15% of the Ki67 positive cells) exhibited γH2AX (data not shown). Consequently it appears that these conditions have still not resulted in sufficient intra-tumoral concentrations to achieve single agent activity with MK-8776 in this xenograft model.

## DISCUSSION

Gemcitabine is used as standard-of-care for the treatment of a wide variety of solid cancers. Its anabolites irreversibly inhibit ribonucleotide reductase and are also incorporated into DNA causing chain termination, either of which cause S phase arrest. DNA damage activates cell cycle checkpoints, and Chk1 inhibitors usually abrogate arrest inducing cell cycle progression and aberrant mitosis [8, 13]. Our data showing that the addition of the Chk1i to S phase-arrested cells increased γH2AX but did not induce cell cycle progression (Figure 6) is explained by the absence of deoxyribonucleotides and thereby suggests that inhibition of ribonucleotide reductase may be the more important mechanism of action of gemcitabine.

Two mechanisms for the resulting cytotoxicity have been proposed. First, it has been suggested that Chk1i treatment of S phase-arrested cells can directly induce mitosis without passage to G_2_, yet this occurred preferentially in p53-defective tumors, and then only in a few of such cell lines [14]. This mechanism cannot explain the fact that Chk1i sensitizes most cancer cell lines to gemcitabine independent of p53 status [11]. A second mechanism proposes that Chk1i enhances replicon firing creating large regions of single-stranded DNA that exceed the capacity of the single-strand DNA binding protein RPA to protect it from nuclease attack; “replication catastrophe” then results [15]. The increase observed in γH2AX in S phase cells upon addition of Chk1i is consistent with replication catastrophe being the most likely mechanism responsible for the efficacy of the combination of gemcitabine plus a Chk1i.

Considering the mechanisms of action of gemcitabine discussed above, the impact of Chk1i to enhance cell killing following gemcitabine treatment would logically be greatest when the majority of cells are arrested in S phase, albeit there is an additional consideration as replication forks continue to evolve for >12 h after arrest to become more Chk1 dependent [11]. We previously demonstrated that administration of Chk1i to mice 18 h after gemcitabine could induce partial tumor regression while administration of the two drugs concurrently had no greater anti-tumor effect than gemcitabine alone [11]. These experiments also showed a significant accumulation of tumor cells in S phase after 18 h. We have now systematically investigated the cell cycle perturbation in cells *in vitro*, in xenograft tumor models, and tumors from bladder cancer patients. Despite the common expectation that the mechanisms observed *in vitro* also occur *in vivo*, we are unaware of any prior studies that have addressed this important question, particularly in cancer patients at drug doses that can be tolerated.

Our initial experiments were designed to compare cytotoxicity of gemcitabine with cell cycle perturbation that occurs in cell lines. There are two important modifications we made to the cytotoxicity assessments. First, we used a brief incubation with gemcitabine (6 h) to more closely parallel the short infusion time when administered to patients. This allows cells time to potentially recover from the drug treatment as occurs in cancer patients. Second, we started our assays with a higher number of cells so that we could observe loss of cell numbers over time. These experiments were also extended to 8 days as it can take many days for cells to die. Gemcitabine is frequently administered to patients on a weekly basis so this data also provides insight into the state of cells before a potential second gemcitabine administration. Using this assay, we were able to establish concentrations that cause “stable disease” *in vitro* (close to no change in cell numbers over 8 days). These concentrations correlated with S phase arrest of at least three days in MDA-MB-231 cells. Higher gemcitabine concentrations resulted in significant numbers of cells with sub-G_1_ DNA content consistent with cell death, and thereby correlating with the cytotoxicity assay. It is also important to note that lower concentrations of gemcitabine caused transient S phase arrest but the cells were able to recover after a few days; these lower concentrations would score as growth inhibition in most cytotoxicity assays despite no apparent cell killing. These were also the cells that we subsequently showed were killed upon addition of MK-8776.

We next performed experiments in AsPC-1 tumors grown as xenografts in mice. This tumor was selected because of our prior demonstration of tumor growth delay *in vivo* [11], and because Ki67 and geminin-stained cells are more homogeneously distributed throughout the tumor. The distribution of Ki67-positive cells is interesting because it is not focal as would occur if there were areas with optimal oxygen or nutrient delivery, rather the positive cells appear randomly distributed and suggest that most cells probably pass through a G_o_ state (Ki67-negative) in each cell cycle. Of the cells progressing through the cell cycle (Ki67-positive), about 40% are in S or G_2_ phase (geminin-positive) in untreated mice. Administration of gemcitabine caused a dramatic accumulation of cells in S and G_2_. Over the range of 40 – 240 mg/kg gemcitabine, most of the Ki67-positive cells also stained positive for geminin after 18 and 24 h. The rate of recovery as observed at 42 and 48 h was slower at the higher gemcitabine doses.

Parallel experiments performed in patients with bladder cancer gave values for cell cycle perturbation similar to that observed in xenografts; all tumors demonstrated that most of the Ki67-positive cells were also positive for geminin after 24 h. Unfortunately, due to changes in local patient referral patterns, we were unable to accrue to a second cohort in which tumors would have been biopsied after 48 h to assess the potential reversibility of the S/G_2_ arrest. The patients in this study received the standard-of-care dose of 1000 mg/m^2^ gemcitabine which is equivalent to 333 mg/kg in mice [16]. Accordingly, the patients received a higher equivalent gemcitabine dose than the mouse studies. The highest dose of gemcitabine used in our mouse studies is considered the maximum tolerated dose. Importantly, much lower doses (40 mg/kg) cause S/G_2_ arrest in murine tumors suggesting that it would be valuable to perform a dose response in patients as this could inform the minimal gemcitabine dose that might be combined with a Chk1i, and consequently might be better tolerated. Lower doses of gemcitabine can also be administered on a more frequent schedule [17], which might provide alternate schedules to test in combination with a Chk1i.

The *in vitro* experiments show that addition of Chk1i to S phase-arrested cells causes a marked increase in γH2AX (Figure 6). Similarly, administration of Chk1i to mice after tumor cells have arrested in S phase also caused a marked increase in γH2AX. Administration of Chk1i at 18 h resulted in more than 50% of the geminin-positive cells being positive for γH2AX. However, when administered at 42 h, far fewer cells were positive for γH2AX suggesting the majority of cells are overcoming the S phase arrest and no longer relying on Chk1 for protection. Our data also show that administration of Chk1i alone had little or no impact on γH2AX. Initially, this was a surprise as the AsPC-1 cells are one of the cell lines most sensitive to MK-8776 as monotherapy *in vitro* [6]. An *in vitro* comparison demonstrated that much higher concentrations and a longer incubation time with Chk1i is required for the monotherapy activity than when combined with gemcitabine. The increased incubation time can be partially explained by the need for all cells to progress into S phase before succumbing to Chk1i while after gemcitabine treatment the S phase proportion has already been enriched. Longer exposure times also increased response to MK-8776 in the combination with gemcitabine. Unfortunately, the short half-life of MK-8776 in human patients [3] probably will not achieve this required drug persistence in tumors after a single bolus, and infusion or multiple injections would be required to achieve improved anti-tumor response.

Merck has discontinued development of MK-8776. However, other Chk1i’s are in preclinical development and clinical trials with promising results, although there is limited information on their bioavailability in mice or man. For example, the Chk1i LY2606368 (prexasertib) when administered as monotherapy twice daily for 3 days, repeated weekly for 4 weeks, resulted in complete regression of sensitive neuroblastoma xenogafts [18]. CCT245737 when administered daily for 9 days caused shrinkage of a myc-driven mouse model of B-cell lymphoma [19]. A Phase I clinical trial with GDC-0425 in combination with gemcitabine demonstrated at least two partial responses [4]. Finally, AZD7762, when combined with irinotecan cause a long-term durable response in one patient, and this was attributed to a loss-of-function mutation in RAD50 [20].

Despite the fact that clinical development of MK-8776 is unlikely to continue, the experimental approaches presented here and the results obtained provide important information for the optimal development of other Chk1i. The results demonstrate the need for determining appropriate dose and schedules, including length of exposure times that should be established preclinically to guide the rational design of subsequent clinical trials. We propose that the assessment of “tumor regression” as seen here in cell culture can be much more informative than the more commonly used assays of growth inhibition. A more detailed treatise on the appropriate design of preclinical experimental design has recently been published [10]. These approaches apply to the development of any other drug alone or in combination and this knowledge will hopefully improve the success rate in clinical trials.

## MATERIALS AND METHODS

### Cell culture

Cell lines were obtained from the American Type Culture Collection, and stored at low passage number as provided. Stock cultures were replaced approximately every 3 months. Cells were maintained in RPMI1640 (Corning/Mediatech) plus 10% fetal bovine serum (Hyclone, Logan UT), and 1% antibiotic/antimycotic (Gibco, Carlsbad, CA).

### Chemicals

MK-8776 was provided by Merck (Kenilworth, NJ); the stock solution was made at 10 mM in dimethylsulfoxide. Gemcitabine in water was obtained from Eli Lilly (Indianapolis, IN) and diluted directly into media. The potency of stock solutions did not change over at least 2 years.

### Cytotoxicity assays

Cells were plated at 10,000/cells per well of a 96-well plate. The following day, drugs were added as required and with various schedules as described in the results (8 wells per concentration). After the treatments, drug was removed, wells washed with phosphate buffered saline, and fresh media added. One plate was harvested at time zero (before drug additions) to provide a value for the starting cell number. Additional plates were harvested every 2 days for 8 days. Plates were washed in phosphate buffered saline and stored at −80°C until analysis. Cells were lysed and DNA was stained with Hoechst 33258 as previously described [13, 21]. Fluorescence was read on a microplate spectrofluorometer.

### Cell cycle analysis

Cell cycle analysis was conducted by flow cytometry using propidium iodide as described previously [11]. For 2-dimensional flow cytometry, cells were also labeled with Alexa 488-conjugated γH2AX (Cell Signaling Technology, Danvers MA). Cells were analyzed on either a Becton Dickinson FACScalibur or Gallios flow cytometer.

### Analysis of tumor xenografts

All animal procedures were performed in strict compliance with the NIH Guide for the Care and Use of Laboratory Animals and approved by the Institutional Animal Care and Use Committee at Dartmouth. To generate tumor xenografts, 2 × 10^6^ AsPC-1 pancreatic cancer cells were injected into the flanks of athymic nu/nu mice. Drug treatments began when the tumors had reached approximately 100 mm^3^. Gemcitabine was administered i.p. at 20 - 240 mg/kg in phosphate buffered saline. MK-8776 was administered i.p. at 50 mg/kg in (2-hydroxypropyl) β-cyclodextrin, 45% w/v solution in water (Sigma). These doses were selected based on our prior publication with these agents [11]. The schedules of administration and times of harvest varied with experiment and are described in the results. At harvest, tumors were fixed in formalin, and serial sections were stained with anti-Ki67 (1:300; Vector Laboratories), anti-geminin (1:200; Santa-Cruz) and anti-γH2AX (1:1500; Cell Signaling Technology) in the Research Pathology Shared Resource. For each tumor, at least 2 fields were photographed, each field representing about 1000 cells; 2-4 individual tumors were scored at each time point. The number of cells positive for each marker were scored by two individuals with the aid of NIH Image J software, and results averaged. The number of cells positive for geminin was expressed as a percentage of those positive for Ki67. Similarly, the number of cells positive for γH2AX were expressed as a percentage of those positive for Ki67.

### Human tumors

This clinical trial was performed with approval of the Dartmouth Committee for the Protection of Human Subjects. Patients diagnosed with muscle-invasive non-metastatic transitional cell bladder carcinoma, and for whom a pre-therapy tumor biopsy was available, were recruited to this proof-of concept clinical study. All enrolled patients gave signed written informed consent prior to any study procedure. The patients were all Caucasian and ranged in age from 61 to 73; four were female and two were male. Patients received 1000 mg/m^2^ gemcitabine i.v. over 30 min, and ∼24 h later underwent a transurethral resection of the bladder cancer. The tumors were fixed in formalin, stained for Ki67 and geminin and scored as for the xenograft tumors above. The primary goal of this trial was to determine whether this standard-of-care dose of gemcitabine induced S/G_2_ phase accumulation of tumor cells as observed in the preclinical tumor xenograft experiments. No adverse events were observed as a result of performing the surgery ∼24 h after administration of gemcitabine. Patients continued off study with standard-of-care gemcitabine alternating with cisplatin/gemcitabine.
